# Retraction Note: Genetic regulation of salt stress tolerance revealed by RNA-Seq in cotton diploid wild species, *Gossypium davidsonii*

**DOI:** 10.1038/s41598-019-45848-y

**Published:** 2019-08-16

**Authors:** Feng Zhang, Guozhong Zhu, Lei Du, Xiaoguang Shang, Chaoze Cheng, Bing Yang, Yan Hu, Caiping Cai, Wangzhen Guo

**Affiliations:** 1 State Key Laboratory of Crop Genetics & Germplasm Enhancement, Hybrid Cotton R & D Engineering Research Center, Ministry of Education, Nanjing Agricultural University, Nanjing, 210095 China; 2 Yunnan Ice Harbor Biotechnology Co. Ltd, Kunming, 650000 China

Retraction of: *Scientific Reports* 10.1038/srep20582, published online 03 February 2016

The authors are retracting this Article.

Data shown in Figure 8C and 8E, and in the Figure 8B and 8F, are duplicated. The authors are unable to provide the original data for these figures, and therefore cannot guarantee the accuracy of these results.

All authors agree with the retraction.

